# Coronavirus disease 2019 (COVID-19) outbreak in Afghanistan: Measures and challenges

**DOI:** 10.1017/ice.2020.240

**Published:** 2020-05-15

**Authors:** Sayed Hamid Mousavi, Milad Abdi, Shafi Ullah Zahid, Kalimullah Wardak

**Affiliations:** 1 Department of the Clinical Biochemistry, Faculty of Medical Sciences, Kateb University, Kabul, Afghanistan; 2 Afghanistan National Charity Organization for Special Diseases, Kabul, Afghanistan; 3 Student Research Committee, Faculty of Medicine, Iran University of Medical Sciences, Tehran, Iran; 4 Department of Microbiology, Faculty of Medicine, Iran University of Medical Sciences, Tehran, Iran; 5 Department of Neurosurgery, Jamhuriat Hospital, Kabul, Afghanistan; 6 Department of Orthopaedics and Traumatology, Wazir Akbar Khan Hospital, Kabul, Afghanistan

*To the Editor—*The new coronavirus (SARS-CoV-2) and the disease it causes (coronavirus disease 2019 (COVID-19) is now a concerning issue that has impacted public health and the global economy. As of May 4, 2020, this infection has involved >210 countries with >3.5 million cases and 240,000 deaths.^1,2^

On February 24, 2020, the first SARS-CoV-2–positive case was reported in Afghanistan in Herat province. The disease has spread rapidly to the major cities and finally to all 34 states in a limited period. As of May 4, 2020, there have been 2,894 SARS-CoV-2–positive cases and 90 COVID-19–related deaths in Afghanistan.^2^

Afghanistan has taken many measures to combat COVID-19, but it also faces a lot of many challenges, many of which fall into 3 areas: prevention, diagnosis, and treatment.^3^

## Prevention

Initially, the Afghanistan Ministry of Interior, which is in charge of police and urban vehicle management, imposed restrictions on travel and gatherings, including banning the Nowruz celebration and other traditional spring gatherings such as poetry and cultural celebrations. After 2 weeks, with the imposition of compulsory quarantine in big cities such as Kabul, Herat, and Mazar-e-Sharif and the traffic restriction on highways leading to provincial centers, the initial restrictions were tightened.^3^

In the early days, the Afghanistan Ministry of Public Health warned that if the ministry’s orders and instructions were not taken seriously, ~80% of people would be infected and thousands would die. It warned that Kabul alone, with a population of ~5 million, must be prepared for >125,000 deaths. Although these high morbidity and mortality rates predicted by the government were not far off the mark, in fact, these harsh warnings were an intimidation strategy. Because most Afghans are accustomed to the daily warnings of the government and the news of death and insecurity, this strategy was used to persuade them to follow health and government instructions.^3^

The first warnings made people panic before the outbreak escalated. The long-standing cultural tradition of shaking hands and hugging was limited, and wearing masks, which had been scorned previously, became somewhat common. Prior to the first COVID-19 case, the World Health Organization (WHO) conducted awareness programs about the disease in villages and towns and mentally prepared the public to cope with the restrictions. Thereafter, the government closed universities, schools, stadiums, wedding halls, public baths, markets, restaurants, malls, and gathering places.^3^

Unfortunately, after a short time, due to the lack of clear statistics and information, severe shortage of protective equipment, food and grains, diagnostic tools, and the dire economic situation, people’s obedience to the government restrictions decreased. As they were forced out of their homes, hygiene and government orders became less effective. Low income, high cost of diagnosis and treatment, inadequate equipment in hospitals, and false rumors prevented people from going to diagnostic centers.^3^ Unfortunately, some clerics encouraged people to engage in daily works and religious activities such as congregational prayers. Due to the support of the people and the tense situation in the country, the government was unable to cope with violations of the infection control and prevention restrictions.

## Diagnosis

To diagnose COVID-19, initially, a diagnosis center was launched only in Kabul. Then, with the increase and spread of this disease in other states including Herat, Balkh, Kandahar, and Nangarhar, subsequent diagnostic laboratories were set up. The relevant centers gradually increased daily tests from 50 to 100 and finally 300 tests.^3^

The shortage of diagnosis kits has been one of the biggest problems in the fight against SARS-CoV-2. Until 4 May 2020, Afghanistan has done ~11,068 diagnostic tests (284 per 1 million population), which is one of the lowest rates among Asian countries.^2^

The high cost of diagnosis, lack of diagnostic kit production, shortage of skilled laboratory staff, inappropriate sampling, many problems in the transfer of samples, including lack of proper roads and low security due to the presence of ISIS and the Taliban, are other important problems preventing the diagnosis of this disease.^3^

## Treatment

To treat SARS-CoV-2–positive cases, the government built medical centers within the diagnostic centers to provide basic services to alleviate these patients’ complications. On a rotating basis, the physicians were assigned to the relevant departments of the public hospitals. The government also hired a number of healthcare workers from the private sector on a contract basis and a few field hospitals were set up in some areas. The WHO, the Jack Ma Foundation, China, India, and other countries have slightly improved the treatment capability by donating protective equipment and ventilators.^3^

Afghanistan is facing a serious shortage of healthcare workers, facilities, and equipment in its treatment response. According to the WHO, Afghanistan is one of the most vulnerable countries in the world, with 9.4 skilled health professionals and 1.9 physicians, per 10,000 population. In Afghanistan, physicians are disproportionately distributed across the country, with 7.2 physicians per 10,000 population in urban areas and only 0.6 physicians per 10,000 population in rural areas.^4^

Approximately 90% of health centers in Afghanistan belong to the private sector, which has not been called upon to diagnose and treat COVID-19. Unfortunately, Afghanistan cannot use this capacity due to the lack of a proper health system, lack of insurance, and high treatment costs. In addition, the lack of protective equipment has also affected many healthcare workers, and some have even been forced to resign or stay home.^3^

Overall, because 71.5% of Afghanistan’s population lives in rural areas, where cinemas, subways, apartment living, and even large public transportation are not common, contact between people is naturally less, which slows the transmission of SARS-CoV-2.^5^ However, other factors, such as young population, long history of BCG vaccination, resistance to harsh and stressful conditions, and exposure to contaminated environments may make the Afghan people more resilient. If the problems are not solved and the situation does not improve, a high percentage of people may be infected and many may die. In addition, this virus may remain in the country for a long time, making it a potential outbreak source in subsequent epidemic waves.

To improve both the present and future situation of Afghanistan to fight COVID-19, we suggest the following measures:

1) Continuing quarantine status in major cities of Afghanistan
2) Increasing the medical equipment and devices for the prevention, diagnosis, and treatment of this disease
3) Increasing the number of and training healthcare workers
4) Informing people through media such as television, radio, web pages, etc
5) Setting up more centers for the diagnosis and treatment in other states
6) Implementing social distancing and more restrictions on travel
7) Strengthening the detection and isolation of patients
8) Closing mosques, congregational prayers, Jumu’ah prayer (Friday prayer), and religious places
9) Providing cash and noncash aid to vulnerable and low-income individuals.

Although Afghanistan has faced many difficulties in prevention, diagnosis, and treatment, implementing these measures may maximize its defense againts COVID-19 pandemic.
